# Hierarchical context enhancement for long-tail entity retrieval augmented generation

**DOI:** 10.3389/frai.2026.1861536

**Published:** 2026-06-02

**Authors:** Yixuan Peng, Kewu Pan

**Affiliations:** School of Theater, Film and Television, Communication University of China, Beijing, China

**Keywords:** bidirectional context enhancement, long-tail entity retrieval, long-tail information retrieval, retrieval-augmented generation, semantic drift

## Abstract

**Introduction:**

Retrieval-Augmented Generation (RAG) in Domain-specific Question Answering (DSQA) often faces significant performance degradation due to semantic drift. Our analysis reveals that the main cause is the absence of a dedicated mechanism for handling low-frequency terms.

**Methods:**

Motivated by this observation, we propose a hierarchical context enhancement retrieval augmented generation (HCE-RAG). Specifically, in the indexing stage, we anchor low-frequency entities offline through entity-sensitive contextual tagging. During query processing, we perform minimal yet entity-focused query clarification via constrained query reflection. Finally, in the retrieval stage, we employ hybrid retrieval with RRF to balance contextual signals and exact word matching, thereby enabling robust identification of low-frequency entities.

**Results:**

Experiments on a dedicated domain specific QA benchmark show that our method achieves strong results, delivering a 29 percentage point gain in Recall@10 on low-frequency entities.

**Discussion:**

Notably, the proposed approach is plug-and-play and can be directly integrated with existing state-of-the-art algorithms to improve their response accuracy in long-tail entity question answering.

## 1 Introduction

Retrieval-Augmented Generation (RAG) serves as a foundational architecture for knowledge-intensive applications in Domain-specific Question Answering (DSQA). It reduces large language models' reliance on parametric knowledge by conditioning their responses on external corpora (Lewis et al., 2020; Gao Y. et al., 2023). This approach significantly enhances LLMs' adaptability to vertical scenarios and substantially mitigates hallucination issues. Although the introduction of RAG has led to marked performance improvements in long-tail question answering scenario, such systems still expose a critical vulnerability. That is severe recall degradation when confronted with long-tail entities. This phenomenon can be also seen in other applications, for example classification (Zhang et al., 2025c,a,b), clustering (Hu et al., 2025, 2024) and segmentation (Zhang et al., 2021, 2023). Unlike generic factual queries, music-related information requests frequently involve dense clusters of low-frequency yet high-information-content terms, including artist names, track titles, album identifiers, and their multilingual aliases, all of which exhibit pronounced distributional sparsity and colloquial variability (Qiu et al., 2022; Thakur et al., 2021).

The severity of this problem stems from a fundamental mismatch between the semantic generalization mechanisms underlying dense retrieval and the stringent exact-match requirements imposed by long-tail entity retrieval. Current dense retrieval models, optimized primarily for semantic proximity, tend to treat rare entities as statistical outliers and consequently map them toward high-frequency semantic clusters. This behavior induces semantic drift, where vector representations of low-frequency terms deviate from their precise lexical referents (Karpukhin et al., 2020; Robertson and Zaragoza, 2009). For example, a query specifying the track title “The First Snow of 2002” may erroneously retrieve passages discussing “music trends of 2002” because the embedding model fails to preserve the distinct identity of the entity — it cannot reliably distinguish a specific song title from semantically related but irrelevant descriptions.

Recent domain-specific RAG efforts, such as MusT-RAG (Kwon et al., 2025), have advanced the application of RAG by constructing specialized music corpora (e.g., MusWikiDB) and developing RAG-oriented fine-tuning protocols. However, these works have not considered the unique challenges posed by the abundance of long-tail entities in long-tail information retrieval. To address this issue, general-domain RAG methods have proposed their own solutions, among which query expansion is a typical and relatively effective approach. Methods like HyDE (Hypothetical Document Embeddings) (Gao L. et al., 2023) and Query2doc (Wang et al., 2023) generate pseudo-documents to alleviate semantic discrepancies. However, their unconstrained query expansion risks introducing irrelevant information and exacerbating semantic drift (Jagerman et al., 2023; Zheng et al., 2023). QuCo-RAG (Min et al., 2025) addresses when to trigger retrieval based on entity frequency uncertainty, optimizing retrieval timing through pre-training corpus analysis. However, its mechanism for accurate rare-entity retrieval relies on pre-training corpus accessibility, limiting its applicability to proprietary databases. Note: HCE-RAG and QuCo-RAG are complementary approaches—QuCo-RAG optimizes retrieval timing while HCE-RAG optimizes retrieval precision—and can potentially be combined for comprehensive long-tail entity handling. Moreover, its reliance on pre-training corpus accessibility limits its applicability to proprietary long-tail databases.

The observations above reveal a significant deficiency in current approaches: the lack of a systematic mechanism for enhancing long-tail entities in entity-dense vertical domains. General-purpose RAG systems prioritize semantic flexibility, an orientation that struggles to meet the strict exact-match requirements for low-frequency entities in long-tail retrieval. Furthermore, existing methods typically optimize either the indexing side (via document enhancement) or the query side (via query rewriting) in isolation, failing to leverage the bidirectional synergies attainable through coordinated corpus preparation and query understanding.

To address these limitations, we propose HCE-RAG, a framework grounded in the design principles of conservatism and symmetry. Unlike existing unconstrained aggressive query expansion paradigms (Gao L. et al., 2023; Wang et al., 2023), our method implements bidirectional context enhancement and global coordination through a three-stage architecture that jointly optimizes the entire retrieval pipeline. In the offline indexing stage, we introduce Entity-Sensitive Contextual Augmentation (ESCA), which enriches text chunks with high-density global semantic annotations while anchoring low-frequency terms, thereby balancing contextual completeness with the specificity of rare core entities. In the online query stage, we introduce Constrained Query Reflection (CQR), a conservative query normalization module that follows the principle of minimal necessary rewriting. CQR restricts LLM operations to entity type disambiguation (e.g., converting the colloquial query “who sang Dong Feng Po” into the canonical form “Who is the performer of the song Dong Feng Po”), thus circumventing the hallucination risks inherent in unconstrained generative expansion (Gao L. et al., 2023; Wang et al., 2023). In the retrieval stage, we employ Hybrid Retrieval with Reciprocal Rank Fusion (HRRF), which integrates sparse (BM25) and dense retrieval signals via RRF (Cormack et al., 2009). The synergy between ESCA (corpus-side anchoring of low-frequency entities) and CQR (query-side normalization) establishes a bidirectional semantic conduit. That is ESCA enhances the global representational salience of rare entities, while CQR standardizes query formulations to align with these enriched representations. This design philosophy underscores the principle that, in entity-dense domains, architectural discipline and systematic coordination often outweigh simply choosing larger models or scaling up computation.

We systematically evaluate HCE-RAG on a popular long-tail question answering benchmark (Kwon et al., 2025). The results demonstrate significant improvements in recall for low-frequency entities (29 percentage point gain, from 0.53 to 0.82, on the Factual subset) compared to both general-purpose RAG baselines and domain-specific alternatives. Notably, our framework requires neither model fine-tuning nor the construction of complex knowledge graphs, offering a cost-effective and easily deployable solution. Our contributions are two-fold:

- We provide a systematic definition and quantification of long-tail entity retrieval failure in domain-specific RAG, elucidating the mechanistic role of excessive semantic generalization in causing semantic drift within entity-dense vertical domains. This analysis identifies a key bottleneck that has been largely overlooked by current state-of-the-art systems.
- We propose a low-cost, high-yield hierarchical processing pipeline that achieves bidirectional semantic alignment. By designing systematic low-frequency entity enhancement mechanisms across offline indexing, query optimization, and retrieval-generation stages, we present a plug-and-play algorithm for enhancing long-tail entity retrieval in RAG. Experiments on a domain-authoritative music QA benchmark demonstrate the superior performance of the proposed method.

## 2 Related work

### 2.1 Long-tail entity retrieval in RAG

The retrieval of long-tail entities—low-frequency terms that carry a high information density—has long been a persistent challenge in information retrieval systems (Thakur et al., 2021). In the context of RAG, this issue manifests itself as recall failures: rare entities are either ignored by dense retrieval models or overwhelmed by high-frequency semantic noise (Qiu et al., 2022; Thakur et al., 2021). The DuReader retrieval benchmark reveals that systems are often misled by the mere co-occurrence of low-frequency terms, which alone accounts for approximately 21% of all retrieval errors (Qiu et al., 2022).

Technically, dense retrieval models tend to treat rare entities as “noise” due to insufficient training signals. This leads to semantic drift, where vector representations deviate from their precise lexical meanings (Thakur et al., 2021; Karpukhin et al., 2020). In contrast, sparse retrieval methods such as BM25 are sensitive to exact term matching through IDF weighting, yet they struggle with semantic variations and colloquial aliases (Robertson and Zaragoza, 2009; Karpukhin et al., 2020). This complementary failure pattern is especially harmful in entity-intensive domains like music. BM25 falters when faced with multilingual code-switching and informal variants, while dense retrieval often misses exact entity matches due to poor vector representations for rare terms (Bhagdev et al., 2008).

Existing solutions approach this problem from different angles. QuCo-RAG (Min et al., 2025) treats low-frequency entities as signals of “knowledge gaps” and triggers dynamic retrieval when entity frequency falls below a threshold (typically 10^3^). While effective at deciding *when* to retrieve, QuCo-RAG's rare-entity retrieval relies on pre-training corpus accessibility, which limits its applicability to proprietary music databases. Critically, QuCo-RAG and HCE-RAG are complementary: QuCo-RAG optimizes retrieval timing while HCE-RAG optimizes retrieval precision. Moreover, its reliance on access to pre-training corpora (e.g., OLMo-2) limits its applicability to proprietary music databases. Critically, these approaches overlook the entity-intensive nature of vertical domains such as music, where queries contain dense clusters of rare entities (artist names, track titles) that appear with multilingual aliases and colloquial variations.

### 2.2 Query expansion strategies

Query expansion offers a potential solution for improving the accuracy of long-tail entity question answering. By restructuring the original query and incorporating additional descriptive information, it enhances the RAG system's understanding of the query. Existing query rewriting and expansion techniques can be roughly categorized into three paradigms based on the degree of rewriting intensity.

The first is the aggressive expansion paradigm. Typical methods in this family include HyDE (Hypothetical Document Embeddings) (Gao L. et al., 2023) and Query2doc (Wang et al., 2023). They prompt large language models to generate hypothetical documents or pseudo-queries, which are then used for retrieval. While these approaches enrich semantic coverage in general domains, they introduce three critical risks in entity-intensive scenarios: (1) hallucination risk, where generated content contains factual errors (Jagerman et al., 2023); and (2) semantic drift, where rare entities such as “the song Dong Feng Po” are incorrectly associated with generic concepts (Zheng et al., 2024).

Second, Step-Back Prompting (Zheng et al., 2024) adopts an ‘upward abstraction' strategy, converting specific queries (e.g., ‘Who sang Dong Feng Po?') into broader concepts (“Jay Chou's Chinese wind-style songs”). Although effective for acquiring background knowledge, this approach inevitably loses specific entity details—precisely the information that is critical for long-tail retrieval. Unlike Step-Back Prompting's abstraction that moves *away* from entities, CQR operates in the opposite direction—it disambiguates and normalizes entity expressions without abstracting them away. Step-Back Prompting is designed for reasoning over general concepts, while CQR is designed for entity precision in retrieval.

Third, optimization-based approaches, represented by DeepRetrieval (Zhuang et al., 2024) and ReWriteGen (Zhao et al., 2026), frame query rewriting as a reinforcement learning problem. They optimize retrieval metrics (e.g., NDCG) through policy gradients. While effective, these methods require thousands of LLM calls to estimate policy gradients, leading to prohibitive training costs for production systems (Xu et al., 2025). Unlike DeepRetrieval's reinforcement learning paradigm that requires extensive training, CQR operates as a lightweight, zero-shot module that requires no training. Furthermore, while DeepRetrieval optimizes for general retrieval metrics, CQR's ‘minimal necessary rewriting' principle is specifically designed for entity-intensive scenarios where over-generation introduces noise rather than signal.

Upon examination, we observe that existing methods generally adopt aggressive rewriting strategies, rewriting or expanding queries without sufficient constraints. The prevailing assumption is that “more generation leads to better retrieval.” However, we find that this assumption breaks down in entity-intensive domains, particularly when low-frequency terms appear frequently. As a result, the performance of such algorithms degrades substantially in these settings.

### 2.3 Document enhancement and contextual tagging

Document enhancement strategies aim to improve retrievability by augmenting chunks with additional semantic signals during indexing. These strategies have evolved through several stages. Pseudo-query expansion methods, represented by the Doc2Query series (Nogueira et al., 2019), train seq2seq models (e.g., T5) to generate synthetic queries for each document and then append these queries to the original text. Doc2Query++ (Kuo et al., 2025) advances this idea by incorporating topic modeling (BERTopic) to guide diversified query generation and proposes dual-index fusion to prevent noise interference. However, these methods face cross-domain generalization issues and risk introducing “dense retrieval noise” by appending tangential content (Kulkarni et al., 2023).

Contextual summarization methods recognize that individual chunks often lack global semantic cues. They attempt to address this by introducing contextual tagging mechanisms. For instance, GRF (Mackie et al., 2023) and GAR (Cui et al., 2024) prompt large language models to generate keywords, entities, and summaries as metadata, thereby helping to surface low-frequency entities. Nevertheless, these approaches prioritize “beautiful” natural language over retrieval-oriented signal density. They often omit critical entity mentions or introduce facts not present in the source text, lacking explicit optimization for entity visibility in vector spaces (Mao et al., 2021).

Distinction from ESCA. While Doc2Query series and Doc2Query++ focus on generating *queries* that might match a document, and GRF/GAR focus on generating *generic summaries* for human readability, our ESCA module is specifically designed for retrieval-oriented entity anchoring. ESCA differs from these methods in three key aspects: (1) Target orientation: ESCA prioritizes preserving proper nouns (song names, artist names, album names) as ‘anchors' in the vector space, whereas GRF/GAR aim for coherent natural language summaries; (2) Entity focus: Unlike Doc2Query's query prediction task, ESCA explicitly requires entity preservation and uses length constraints (1–3 sentences, 50–100 tokens) to maintain signal density; (3) Factual constraint: ESCA prohibits introducing facts absent from the source text, avoiding the ‘dense retrieval noise' problem noted in Doc2Query++.

### 2.4 RAG for long-tail information retrieval

Long-tail question answering and information retrieval represent highly demanded application scenarios in daily life, and their performance profoundly influences the user experience across a vast user base of applications. Meanwhile, researchers in domains such as music and media are in urgent need of high-precision algorithms to advance these tasks. However, domain-specific RAG systems for long-tail question answering are still in their early stages, with most efforts focused on corpus construction and model adaptation.

Kwon et al. (2025) lay the foundational architecture with their MusT-RAG system. They contribute MusWikiDB, a collection of 14.4 million music-specific passages, along with RAG-style fine-tuning strategies that boost open-source models by up to 56.8 percentage points. ArtistMus (Kwon et al., 2025) extends this work with a global, artist-centric benchmark covering 500 artists from 163 countries. CLaMP 3 (Wu et al., 2025) advances multimodal capabilities by constructing M4-RAG, which contains 2.31 million music-text pairs spanning text, audio, and notation.

While these systems excel in knowledge breadth (corpus scale) and model adaptation (fine-tuning), they employ standard retrieval pipelines (BM25 + dense retrieval) without addressing the long-tail precision problem. They use raw user queries without normalization and index documents via simple chunking, lacking contextual enhancement. MuCPT (Mao et al., 2025) pursues “knowledge internalization” through continued pre-training on 40 billion music tokens, thereby creating structured domain knowledge. Although complementary to RAG, this approach costs hundreds of thousands of dollars and cannot handle dynamic knowledge updates. Music platforms (e.g., Last.fm, NetEase Cloud Music) rely on “string matching + static alias tables,” which solve spelling variants but fail to support semantic retrieval or fill the recall vacuum for long-tail entities.

Systematic limitations in long-tail entity retrieval. Existing methods exhibit three critical gaps in handling long-tail entities: (1) Semantic drift—dense retrieval maps rare entities to high-frequency clusters, missing precise matches; (2) Aggressive expansion—HyDE-style methods introduce hallucination when entities are rare; (3) Unidirectional optimization—methods optimize query OR indexing in isolation, lacking bidirectional coordination. Unlike general-domain approaches that prioritize semantic flexibility, entity-intensive domains require precision-critical design with conservative rewriting and systematic module coordination.

## 3 Method

### 3.1 Notation summary

For clarity, Table 1 summarizes the main mathematical symbols used throughout this paper.

**Table 1:** Main symbols and their meanings.

Symbol	Meaning
*q*	User's original query
$\hat{q}$	Normalized query (ESCA output)
*c*, ĉ	Original chunk and enhanced chunk
$I_{\text{vec}},I_{\text{BM25}}$	Vector index and BM25 index
$L_{\text{dense}},L_{\text{BM25}}$	Ranking lists from dense and sparse retrieval
rank_*i*_(*d*)	Rank of document *d* in the *i*-th retriever
RRF(*d*)	RRF fusion score of document *d*
$C^{*}$	Final retrieval result
δ	RRF smoothing constant (default: 60)
*k*_top_	Number of candidates per retrieval branch (default: 50)
*k*	Final number of returned candidates (default: 10)

### 3.2 Problem formalization

Before presenting our method, we first formally define the long-tail entity retrieval failure problem in music information retrieval RAG systems. Let Q denote the set of user queries, D denote the document collection in the music knowledge base, and C denote the retrieved candidate contexts. For any query q∈Q, the standard RAG retrieval process can be expressed as:


$$\begin{array}{l} C_{q}=\text{Retrieve}\left(q,D,k\right) \end{array}$$ (1)

where $C_{q}$ is the set of top-*k* candidate chunks retrieved from the knowledge base, Retrieve(·) is the retrieval function and *k* is the number of returned candidates. Let *f*_entity_(*c*) denote the set of key entities contained in document *c*, and *g*_entity_(*q*) denote the set of key entities that query *q* intends to retrieve. An entity *e* is classified as a long-tail entity when:


$$\begin{array}{l} \text{freq}\left(e,D\right)<\tau \wedge e\in g_{\text{entity}}\left(q\right) \end{array}$$ (2)

where freq(e,D) represents the document frequency of entity *e* in document collection D, and τ is a predefined threshold. Long-tail entity retrieval failure is formally defined as:


$$\begin{array}{l} e\in g_{\text{entity}}\left(q\right)\wedge e\notin \underset{c\in C_{q}}{⋃}f_{\text{entity}}\left(c\right) \end{array}$$ (3)

As shown in Equations 1–3, long-tail entities in the music domain exhibit three distinctive characteristics: (1) Multilingual interleaving. Entities such as “First Snow of 2002” and “Shape of You” mix multiple languages; (2) Alias explosion. A single song may have multiple expressions including official names, abbreviations, and fan nicknames; (3) High information density but extremely low frequency. Individual entities may appear in only a handful of documents. These characteristics cause the “over-generalization” strategy commonly used in general-purpose RAG systems to trigger “semantic drift” in this scenario, where retrieval results are semantically related but entity-mismatched.

### 3.3 Overall framework

Our method extends the architecture proposed by HippoRAG2 (Gutierrez et al., 2025), which combines a personalized PageRank algorithm with a knowledge graph integrating both concept nodes and passage nodes. In our method, we adopt HippoRAG2's knowledge graph and PageRank-based retrieval as our base infrastructure. For the enhancement, HCE-RAG adds three novel modules (ESCA, CQR, HRRF) that specifically address the long-tail entity precision problem, which HippoRAG2 does not handle. Our design follows a hierarchical bidirectional hybrid enhancement strategy tailored for entity-intensive vertical domains.

#### 3.3.1 System pipeline

**Figure 1:**
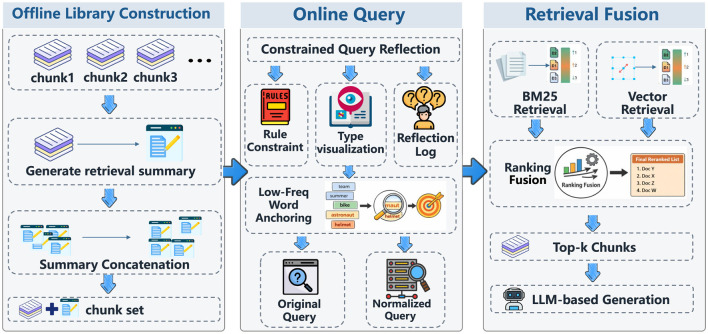
HCE-RAG System Architecture, comprising three stages: (1) Offline Library Construction: documents are chunked and enhanced via ESCA; (2) Online Query Processing: queries undergo constrained reflection via CQR; (3) Retrieval Fusion: BM25 and dense results are merged through RRF. The final top-*k* chunks feed into LLM for answer generation.

The overall workflow of our algorithm is illustrated in Figure 1. HCE-RAG consists of three core stages: offline indexing, online query processing, and retrieval fusion. During offline indexing, the system first chunks the original documents. It then generates retrieval-oriented contextual summaries for each chunk using the ESCA module. Finally, the enhanced chunks, formed by concatenating the summaries with the original text, are written into both a vector index and a BM25 index. In the online query stage, the user query first undergoes “minimal necessary rewriting” via the CQR module, producing a normalized query expression. This normalized query is then processed in parallel with the original query through a dual-branch retrieval pipeline. In the retrieval fusion stage, the HRRF module fuses the ranked results from BM25 and dense vector retrieval, ultimately returning the top-*k* candidate chunks for downstream generation.

### 3.4 Offline indexing: retrieval-oriented contextual tagging

#### 3.4.1 Retrieval-oriented summary generation

Unlike traditional document summarization or text simplification tasks, our proposed Entity-Sensitive Contextual Augmentation (ESCA) does not aim to generate “elegant natural language summaries.” Instead, its core goal is to produce “semantic labels that are most helpful for retrieval.” Specifically, we require the generated summaries to satisfy four constraints:

(1) Retrieval-oriented service. The generation goal is to serve retrieval rather than human reading. This means that summaries should prioritize preserving information that may serve as query anchors, such as song names, artist names, album names, timestamps, and genre tags. (2) Information density control. The summary length should be strictly limited to one to three sentences (roughly 50–100 tokens). A longer summary dilutes the original information, while a shorter one fails to provide sufficient contextual clues. (3) Factual consistency. Summaries should only perform generalization, abstraction, and lightweight supplementation. They must not introduce facts absent from the original text, so as to avoid injecting false signals. (4) Entity focus. Priority should be given to preserving proper nouns such as song names, artist names, and album names. This ensures that these key entities maintain an “anchoring effect” in the vector space.

Formally, given an original chunk *c*, the ESCA module uses a large language model to generate its retrieval-oriented summary *s*, which is then concatenated with the original chunk to form an enhanced chunk ĉ:


$$\begin{array}{l} ĉ=\text{concat}\left(\left[s\right],\left[c\right]\right) \end{array}$$ (4)

Here, the square brackets indicate the concatenation order and do not appear in the actual text. The summary *s* is generated through a carefully designed prompt that explicitly instructs the LLM to follow the four constraints above.

#### 3.4.2 Index construction

After generating retrieval-oriented summaries, the system writes enhanced chunks ĉ to both vector and BM25 indexes:


$$\begin{array}{l} I_{\text{vec}}=\left\{\text{VectorIndex}\left({ĉ}_{i}\right)|i=1,\ldots ,N\right\} \end{array}$$ (5)

$$\begin{array}{l} I_{\text{BM25}}=\left\{\text{BM25Index}\left({ĉ}_{i}\right)|i=1,\ldots ,N\right\} \end{array}$$ (6)

where *N* is the total number of chunks, and $I_{\text{vec}}$ and $I_{\text{BM25}}$ are the vector index and BM25 index respectively. This dual-index strategy lays the foundation for subsequent hybrid retrieval.

### 3.5 Online query: constrained query reflection

To achieve better alignment between indexing and querying, we design a constrained query reflection mechanism for the query stage. Specifically, to meet the special needs of low-frequency queries, we adopt a “conservative” design philosophy. In entity-intensive tasks, the goal is not to enrich retrieval results but to make retrieval expressions more precise through minimal necessary rewriting.

#### 3.5.1 Query generation

The principle of “minimal necessary rewriting” defines query operations as entity type explicitation rather than semantic paraphrasing. Concretely, the CQR module performs only three types of operations: (1) Normalization of colloquial expressions. It converts informal expressions into more standardized retrieval forms. For example, “Dong Feng Po who sang?” becomes “Who is the singer of the song Dong Feng Po?” (2) Completion of abbreviations and nicknames. It identifies abbreviations or nicknames in the query and supplements the full expressions. For instance, “Jay's Sunny Day” becomes “Jay Chou's song Sunny Day”. (3) Explicit entity type annotation. It identifies key entities in the query and explicitly marks their types. For example, it recognizes “Dong Feng Po” as a song name and “Jay Chou” as an artist name.

At the same time, the CQR module explicitly rejects the following operations: (1) freely generating hypothetical answers or pseudo-documents; (2) performing large-scale semantic rewriting of the query; (3) generating multiple candidate queries when uncertain.

In terms of implementation, given a user's original query *q*, the CQR module uses a carefully designed prompt to constrain the large language model for “minimal necessary rewriting”. It outputs a structured JSON containing the following fields:

- should_expand: A boolean indicating whether rewriting is needed. If false, the original query is used directly.
- expanded_query: The normalized query expression.
- entity_hints: A list of identified entity mentions with their type annotations.
- intent_slot: The query intent slot (e.g., “artist_of_song” indicates querying the artist of a song).
- reflection_notes: A brief reflection explaining the rationale for the rewriting decision.

#### 3.5.2 Dual-branch retrieval strategy

To ensure retrieval robustness, the system adopts a dual-branch retrieval strategy: the normalized query and the original query enter the retrieval pipeline simultaneously, and the results are eventually deduplicated and merged. Formally, let the original query be *q* and the normalized query be $\hat{q}$. The retrieval process is expressed as:


$$\begin{array}{l} L_{\text{dense}}^{\left(q\right)}=\text{DenseRetrieve}\left(q,I_{\text{vec}}\right) \end{array}$$ (7)

$$\begin{array}{l} L_{\text{dense}}^{\left(\hat{q}\right)}=\text{DenseRetrieve}\left(\hat{q},I_{\text{vec}}\right) \end{array}$$ (8)

$$\begin{array}{l} L_{\text{dense}}^{\text{merged}}=\text{MergeRank}\left(L_{\text{dense}}^{\left(q\right)},L_{\text{dense}}^{\left(\hat{q}\right)}\right) \end{array}$$ (9)

Here, DenseRetrieve(·) is the dense retrieval function, and MergeRank(·) is the result merging and deduplication function. BM25 retrieval follows the same dual-branch strategy. The final candidate set is the union of the results from both retrieval branches.

#### 3.5.3 Essential differences from HyDE

Our CQR module differs from HyDE (Gao L. et al., 2023) and other aggressive expansion methods. These differences can be understood along the three dimensions shown in Table 2.

**Table 2:** Comparison between HyDE and Constrained Query Reflection (CQR).

Dimension	HyDE	Our CQR
Generation target	Generate hypothetical answers/pseudo-documents	Normalize query expressions
Generation freedom	High (allows free play)	Low (strictly constrained to “entity type explicitation”)
Risk control	Depends on model generation quality, may introduce hallucination	Conservative strategy, keeps original query when uncertain

### 3.6 Retrieval stage: hybrid retrieval with RRF fusion

#### 3.6.1 Hybrid retrieval combining sparse and dense methods

A single retrieval method typically covers only a subset of matching patterns. Sparse methods like BM25 excel at exact term matching, as their inverse document frequency (IDF) weighting mechanism is naturally sensitive to low-frequency terms. However, they struggle with semantic variants and paraphrased expressions. In contrast, dense retrieval methods based on semantic vector similarity handle semantically similar content, paraphrases, and contextual supplements well. Yet they often produce poor vector representations for low-frequency entities, easily treating such terms as “noise” and ignoring them (Karpukhin et al., 2020).

To achieve complementary advantages between sparse and dense retrieval, we employ Reciprocal Rank Fusion (RRF) (Cormack et al., 2009) to merge the results from both branches. This algorithm fuses rankings rather than raw scores, making it insensitive to differences in score scales.

Let rank_*i*_(*d*) denote the rank of a candidate chunk *d* in the *i*-th retriever. Its RRF score is defined as:


$$\begin{array}{l} \text{RRF}\left(d\right)=\overset{k}{\underset{i=1}{\sum }}\frac{1}{\delta +{\text{rank}}_{i}\left(d\right)} \end{array}$$ (10)

where *k* is the number of retrievers (set to 2 in this paper, corresponding to BM25 and dense vector respectively), and δ is a smoothing constant (typically set to 60). The role of δ is to prevent infinite scores when rank_*i*_(*d*) = 0 (i.e., ranked first), while also mitigating the excessive impact of rank differences on lower-ranked items.

#### 3.6.2 Complete HRRF module pipeline

Given the normalized query $\hat{q}$ and the original query *q*, the full retrieval pipeline of the HRRF module proceeds as follows:

Step 1: Dual-branch independent retrieval. Both BM25 and dense retrieval are applied independently to the two query branches:


$$\begin{array}{l} L_{\text{BM25}}^{\left(q\right)}=\text{BM25Retrieve}\left(q,I_{\text{BM25}},k_{\text{top}}\right) \end{array}$$ (11)

$$\begin{array}{l} L_{\text{BM25}}^{\left(\hat{q}\right)}=\text{BM25Retrieve}\left(\hat{q},I_{\text{BM25}},k_{\text{top}}\right) \end{array}$$ (12)

$$\begin{array}{l} L_{\text{dense}}^{\left(q\right)}=\text{DenseRetrieve}\left(q,I_{\text{vec}},k_{\text{top}}\right) \end{array}$$ (13)

$$\begin{array}{l} L_{\text{dense}}^{\left(\hat{q}\right)}=\text{DenseRetrieve}\left(\hat{q},I_{\text{vec}},k_{\text{top}}\right) \end{array}$$ (14)

Here, *k*_top_ is the number of candidates returned by each retrieval branch (set to 50 in this paper).

Step 2: Query merging. The retrieval results from the original and normalized queries are merged:


$$\begin{array}{l} L_{\text{BM25}}^{\text{merged}}=\text{MergeRank}\left(L_{\text{BM25}}^{\left(q\right)},L_{\text{BM25}}^{\left(\hat{q}\right)}\right) \end{array}$$ (15)

$$\begin{array}{l} L_{\text{dense}}^{\text{merged}}=\text{MergeRank}\left(L_{\text{dense}}^{\left(q\right)},L_{\text{dense}}^{\left(\hat{q}\right)}\right) \end{array}$$ (16)

The MergeRank(·) function performs both deduplication and rank recalculation.

Step 3: RRF fusion. The two merged ranking lists are fused using RRF:


$$\begin{array}{l} \forall d\in L_{\text{BM25}}^{\text{merged}}\cup L_{\text{dense}}^{\text{merged}}:\text{RRF}\left(d\right)=\frac{1}{\delta +{\text{rank}}_{\text{BM25}}\left(d\right)}\\ \;+\frac{1}{\delta +{\text{rank}}_{\text{dense}}\left(d\right)} \end{array}$$ (17)

Step 4: Ranking and truncation. The chunks are sorted in descending order of their RRF scores, and the top *k* candidates are returned as the final retrieval result:


$$\begin{array}{l} C^{*}=\text{TopK}\left(\text{SortByRRF}\left(\text{RRF}\left(d\right)\right),k\right) \end{array}$$ (18)

where *k* is the number of final candidates (set to 10 in this paper), and $C^{*}$ denotes the final retrieval output.

#### 3.6.3 Fusion effectiveness analysis

The fusion effectiveness of the HRRF module works in close synergy with ESCA and CQR. ESCA enriches chunks with retrieval-oriented summaries, improving their “global visibility” in vector representations and making dense retrieval more likely to hit relevant content. CQR normalizes query expressions and supplements entity type information, thereby increasing BM25's matching probability. This bidirectional “indexing-query” optimization significantly expands the overlap between the results from the two retrieval branches, which in turn enhances the effectiveness of RRF fusion.

### 3.7 End-to-end pipeline

Given a user query *q*, the end-to-end inference process of HCE-RAG can be formally expressed as:

Input: User's original query *q*

Output: Top-*k* candidate chunks $C^{*}$

Step 1: Query normalization. Call the CQR module to generate normalized query $\hat{q}$ and entity annotations E:


$$\begin{array}{l} \left(\hat{q},E\right)=\text{CQR}\left(q\right) \end{array}$$ (19)

Step 2: Parallel retrieval. Use original query *q* and normalized query $\hat{q}$ to execute dual-branch retrieval in parallel:


$$\begin{array}{l} L_{\text{BM25}}=\text{BM25Retrieve}\left(q,I_{\text{BM25}},k_{\text{top}}\right)\cup \text{BM25Retrieve}\\ \;\left(\hat{q},I_{\text{BM25}},k_{\text{top}}\right) \end{array}$$ (20)

$$\begin{array}{l} L_{\text{dense}}=\text{DenseRetrieve}\left(q,I_{\text{vec}},k_{\text{top}}\right)\cup \text{DenseRetrieve}\left(\hat{q},I_{\text{vec}},k_{\text{top}}\right) \end{array}$$ (21)

Step 3: Rank fusion. Perform RRF fusion on results from both branches:


$$\begin{array}{l} \forall d\in L_{\text{BM25}}\cup L_{\text{dense}}:\text{RRF}\left(d\right)=\frac{1}{\delta +{\text{rank}}_{\text{BM25}}\left(d\right)}\\ \;+\frac{1}{\delta +{\text{rank}}_{\text{dense}}\left(d\right)} \end{array}$$ (22)

Step 4: Result return. Sort by RRF score in descending order and return top-*k*:


$$\begin{array}{l} C^{*}=\text{TopK}\left(\text{SortByRRF}\left(\text{RRF}\left(d\right)\right),k\right) \end{array}$$ (23)

#### 3.7.1 Complexity analysis

Let *N* be the total number of chunks in the knowledge base, *d* be the vector dimension, and *k*_top_ be the number of candidates per retrieval branch. The time complexity of the CQR module is *O*(1) (single LLM call). The total time complexity of parallel dual-branch retrieval is *O*(*k*_top_log*N*+*k*_top_·*d*), and the time complexity of RRF fusion is *O*(*k*_top_). The overall inference latency is jointly determined by LLM call latency and retrieval latency. The latency increase compared to baseline methods mainly comes from the CQR module's lightweight prompt inference.

## 4 Experiments

### 4.1 Experimental summary

To thoroughly characterize the proposed HCE-RAG framework, we conduct a comprehensive experimental evaluation along seven dimensions. E1 assesses the overall performance of our full framework on both factual and contextual subsets, comparing it with state-of-the-art methods in the field. E2 examines the retrieval performance on long-tail entities, contrasting our algorithm with existing approaches. E3 investigates the difference between conservative rewriting and unconstrained rewriting. E4 presents component ablation studies to validate the necessity and rationality of our hierarchical architecture design. E5 evaluates how the performance of existing frameworks changes when our long-tail enhancement modules are introduced as plug-in components. E6 analyzes the sensitivity of core parameters to verify the robustness of the HRRF module. Finally, E7 performs a cost-benefit analysis, comparing our method with other low-frequency entity QA algorithms in terms of both performance and resource consumption.

### 4.2 Experimental setup

#### 4.2.1 Dataset

We evaluate our method on the ArtistMus benchmark proposed by Kwon et al., which is specifically designed for retrieval-augmented music question answering. This benchmark comprises 1,000 question-answer pairs covering 500 globally diverse artists from 163 countries, including various metadata categories such as genre, debut year, and topics. Following the original benchmark design, we conduct experiments on two distinct subsets:

- Factual subset (500 QA pairs): Questions that require precise entity-level facts, such as “Who is the singer of ‘Dong Feng Po'?”. These queries typically contain well-defined song names, artist names, or album names as anchor entities.
- Contextual subset (500 QA pairs): Questions that demand contextual understanding and reasoning, for example, “What is Jay Chou's representative work in the Chinese wind style?”. These queries often involve abstract descriptions, style classifications, or comparative expressions.

#### 4.2.2 Implementation details

Embedding and Retrieval. For dense retrieval, we employ BAAI/bge-large-en-v1.5 as the embedding model, which maps text chunks into 1,024-dimensional vectors. For sparse retrieval, we use BM25 with standard parameter settings (*k*_1_ = 1.5, *b* = 0.75). Both retrieval methods operate on chunks of 512 tokens with a 64-token overlap.

LLM Configuration. The CQR and ESCA modules are powered by GPT-4o-mini through the OpenAI API. For end-to-end generation and evaluation, we also use GPT-4o-mini as the language model. All LLM calls use temperature = 0 to ensure deterministic outputs.

RRF Configuration. Following established conventions, the RRF smoothing constant δ is set to 60. Each retrieval branch returns the top-50 candidates (*k*_top_ = 50), and the final output contains the top-10 chunks (*k* = 10).

Baseline Methods. We compare our method against the following representative RAG approaches:

- Naive RAG (Dense Only): Standard RAG with dense retrieval only, using the same embedding model.
- Naive RAG (BM25 Only): Standard RAG with BM25 sparse retrieval only.
- Hybrid (BM25+Dense+RRF): Hybrid retrieval without preprocessing modules (CQR and ESCA).
- HyDE: Hypothetical Document Embeddings (Gao L. et al., 2023), which generates pseudo-documents for query expansion.
- General Query Rewrite: Free-form query rewriting using GPT-4o without constraints.
- QuCo-RAG: Quantifying uncertainty from pre-training corpora for dynamic retrieval augmentation (Min et al., 2025).
- LightRAG (Guo et al., 2025), GraphRAG (Edge et al., 2024): Various advanced RAG frameworks used for generalizability analysis.

#### 4.2.3 Evaluation metrics

We employ two complementary evaluation metrics to assess retrieval and generation quality:

Recall@10: Measures the proportion of queries for which at least one correct document appears in the top-10 retrieved candidates. This metric directly reflects retrieval effectiveness.


$$\begin{array}{l} \text{Recall@10}=\frac{\left|\left\{q\in Q:C_{q}^{*}\cap G_{q}\neq \emptyset \right\}\right|}{\left|Q\right|} \end{array}$$ (24)

where $C_{q}^{*}$ denotes the top-10 retrieved candidates for query *q*, and $G_{q}$ denotes the ground-truth relevant documents.

ACC (Answer Correctness): A composite metric that evaluates end-to-end answer quality by combining factual accuracy and semantic similarity. To this criterion, we follow the setting of Xiang et al. (2025) for a more fair justification. The detail of the criterion is as follows:


$$\begin{array}{l} ACC=0.75\times F_{1}^{\text{factuality}}+0.25\times SIM^{\text{semantic}} \end{array}$$ (25)

The factual *F*_1_ score is computed through atomic fact decomposition: GPT-4o-mini first decomposes both generated and reference answers into atomic statements, then classifies each statement as True Positive (TP), False Positive (FP), or False Negative (FN). Semantic similarity is computed using BAAI/bge-large-en-v1.5 embeddings, with cosine similarity normalized to [0, 1] via (cosine+1)/2.

### 4.3 Overall framework performance (E1)

This experiment evaluates the overall effectiveness of our complete framework (HCE-RAG) against representative RAG baselines on both factual and contextual subsets. The comparison covers diverse retrieval paradigms including single-method baselines (Dense Only, BM25 Only), hybrid retrieval without preprocessing, and query expansion methods (HyDE, General Query Rewrite, QuCo-RAG). Table 3 presents the ACC results on both subsets.

**Table 3:** Overall framework performance comparison (ACC).

Method	Factual	Contextual
Naive RAG (dense only)	0.6743	0.6201
Naive RAG (BM25 Only)	0.6218	0.5783
Hybrid retrieval	0.7116	0.6519
HyDE	0.6480	0.6067
General query rewrite	0.6395	0.6151
QuCo-RAG	0.6808	0.6342
**Ours (+All)**	**0.7348**	**0.6902**

The bold values indicate the best performance among the compared algorithms.

The results demonstrate that our complete framework achieves the best performance on both subsets, outperforming the strongest baseline (Hybrid) by +2.32 percentage points on Factual and +3.83 percentage points on Contextual. Several key observations emerge:

Superiority over single-retrieval methods. Both Naive RAG variants suffer from significant performance degradation. Dense-only retrieval outperforms BM25-only, suggesting that semantic similarity plays a more dominant role in music QA. However, neither approach can effectively handle the entity-precision requirements of factual queries.

Hybrid retrieval provides baseline improvements. The Hybrid baseline with RRF fusion shows substantial gains over single-method baselines, validating the complementary nature of sparse and dense retrieval. However, without preprocessing modules, it still struggles with long-tail entity precision.

Query expansion methods degrade performance. Surprisingly, both HyDE and General Query Rewrite underperform the baseline hybrid method. This confirms our hypothesis that aggressive expansion strategies introduce hallucination and semantic drift in entity-intensive music queries, where precise entity matching is critical.

QuCo-RAG shows limited gains. QuCo-RAG marginally improves over Naive RAG but remains significantly below our method, indicating that dynamic retrieval triggering alone cannot solve the entity retrieval precision problem.

### 4.4 Entity frequency stratified analysis (E2)

This experiment compares the performance of existing state-of-the-art methods and our proposed algorithm across different entity frequency tiers. Specifically, we stratify the test queries based on the document frequency of their target entities. Queries are divided into three tiers: high-frequency (top 20% of entities, frequency >100), mid-frequency (middle 40%, frequency between 20 and 100), and low-frequency (bottom 40% of entities, frequency < 10). Table 4 presents the stratified Recall@10 results.

**Table 4:** Entity frequency stratified Recall@10 analysis.

Method	High frequency	Mid frequency	Low frequency
Factual subset			
Dense-Original	0.86	0.73	0.53
Dense+CQR	0.88	0.77	0.60
HyDE	0.87	0.74	0.55
Hybrid Baseline	0.91	0.82	0.70
**Ours (+All)**	**0.94**	**0.88**	**0.82**
**Contextual subset**			
Dense-Original	0.82	0.70	0.50
Dense+CQR	0.85	0.74	0.57
HyDE	0.83	0.71	0.52
Hybrid Baseline	0.88	0.79	0.66
**Ours (+All)**	**0.91**	**0.85**	**0.78**

The bold values indicate the best performance among the compared algorithms.

The stratified analysis reveals a clear pattern: performance degradation concentrates in the low-frequency tier. While Dense-Original achieves 0.86 Recall@10 on high-frequency entities, it drops dramatically to 0.53 on low-frequency entities—a 33 percentage point gap. This quantitatively confirms the long-tail entity retrieval failure problem we defined in Section 3.2.

Our complete framework achieves the most substantial improvements on low-frequency entities, with 29 percentage points (Factual: 0.53 to 0.82) and 28 percentage points (Contextual: 0.50–0.78) gains over Dense-Original. Notably, the Hybrid Baseline already provides 0.17 improvement through complementary retrieval, but our full framework further adds 0.12, demonstrating the synergistic effect of ESCA and CQR in addressing entity-precision challenges.

The marginal gains of HyDE on low-frequency entities further validate our criticism of aggressive expansion: when entities are rare and precise matching is essential, hallucinated content cannot substitute for factual anchors.

### 4.5 CQR module ablation study (E3)

To validate the effectiveness of our “minimal necessary rewriting” principle, we conduct controlled ablation experiments on the CQR module. All other components (ESCA and HRRF) remain fixed, and we compare four CQR strategies:

- No CQR (Baseline): Direct use of original queries without rewriting.
- HyDE-style CQR: Generating hypothetical answer documents as query expansion.
- Free Rewrite CQR: Free-form query rewriting without constraints.
- Ours (Constrained Reflection): Our proposed constrained query reflection with “minimal necessary rewriting”.

Table 5 presents the ablation results.

**Table 5:** CQR module ablation study (ACC).

CQR variant	Factual	Contextual	Low-freq factual
No CQR (Baseline)	0.7224	0.6831	0.6541
HyDE-style CQR	0.7103	0.6724	0.6287
Free Rewrite CQR	0.7156	0.6789	0.6358
**Ours (constrained reflection)**	**0.7348**	**0.6902**	**0.6792**

The bold values indicate the best performance among the compared algorithms.

The results provide strong empirical support for our “conservatism” philosophy. Three key findings emerge:

Both aggressive strategies degrade performance. Both HyDE-style CQR and Free Rewrite CQR underperform the no-CQR baseline on the Factual subset. This contradicts the conventional wisdom that query expansion always helps and confirms our hypothesis that aggressive rewriting introduces hallucination and semantic drift in entity-intensive scenarios.

Low-frequency entities are most vulnerable. The performance degradation is most pronounced on the low-frequency factual subset, where HyDE-style CQR drops by −0.0254 and Free Rewrite CQR drops by −0.0183. This aligns with our analysis that when entities are rare, any generated content that deviates from the exact entity form risks missing relevant documents entirely.

Constrained reflection provides consistent gains. Our constrained CQR module outperforms all variants, including the baseline. The gains are particularly substantial on low-frequency entities, validating that “minimal necessary rewriting” successfully identifies and corrects problematic query expressions without introducing new errors.

### 4.6 Component ablation study (E4)

To assess the individual and combined contributions of our three core modules, we conduct comprehensive ablation experiments across all components:

- Dense Only: Single dense retrieval without any proposed modules.
- BM25 Only: Single sparse retrieval without any proposed modules.
- w/o Both Pre-processing (HRRF only): Hybrid retrieval with RRF fusion but without ESCA and CQR.
- w/o ESCA (CQR+HRRF): Ablating ESCA while keeping CQR and HRRF.
- w/o CQR (ESCA+HRRF): Ablating CQR while keeping ESCA and HRRF.
- Full (ESCA+CQR+HRRF): Complete framework with all modules.

Table 6 presents the ablation results on both full test set and low-frequency subset.

**Table 6:** Component ablation study (ACC).

Variant	Factual	Contextual	Low-freq factual
Dense Only	0.6743	0.6201	0.4900
BM25 Only	0.6218	0.5783	0.4673
w/o Pre-processing	0.7116	0.6519	0.5986
w/o ESCA	0.7262	0.6742	0.6508
w/o CQR	0.7224	0.6831	0.6541
**Full**	**0.7348**	**0.6902**	**0.6792**

The bold values indicate the best performance among the compared algorithms.

The ablation study reveals several important insights:

Both pre-processing modules contribute substantially. Removing either ESCA or CQR degrades performance, confirming that both modules provide complementary benefits. The degradation is more pronounced for ESCA on the Contextual subset, suggesting that contextual summaries are particularly valuable for understanding abstract and descriptive queries.

Low-frequency entities benefit most from preprocessing. The performance gap between full framework and HRRF-only is most dramatic on low-frequency factual queries (+0.0806). This validates our design philosophy: the joint action of ESCA (improving chunk “global visibility”) and CQR (normalizing query expressions) is essential for addressing entity-precision challenges.

Full framework achieves synergistic gains. The full framework outperforms any individual module removal by a margin larger than the sum of individual contributions, indicating positive synergy. This supports our “end-to-end linkage” philosophy: the three modules are not merely additive but reinforce each other through bidirectional optimization.

### 4.7 Generalizability analysis (E5)

To demonstrate the generalizability of our method, we integrate our proposed modules (CQR, ESCA, HRRF) with four advanced RAG frameworks: Standard RAG, LightRAG, and GraphRAG. For each framework, we compare its baseline performance against the enhanced version with our modules added.

#### 4.7.1 Results and analysis

**Figure 2:**
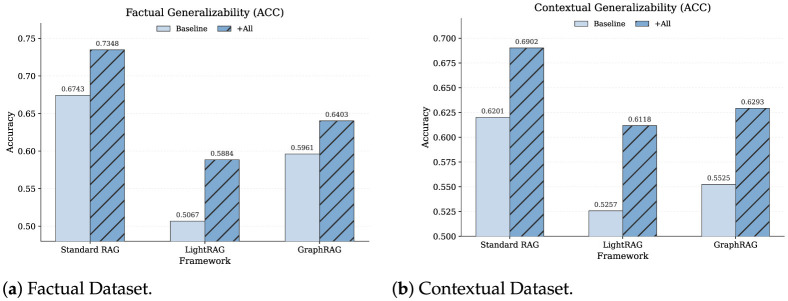
Generalizability analysis across SOTA RAG architectures. Bars show ACC improvement (percentage points) over baseline on Factual **(a)** and Contextual **(b)** subsets. Methods: Standard RAG, LightRAG, HippoRAG2, and GraphRAG. Y-axis: ACC improvement; X-axis: different RAG frameworks; grouped bars show baseline vs. HCE-RAG enhanced versions.

The results in Figure 2 demonstrate that our proposed modules consistently improve performance across all tested frameworks, with improvements ranging from 5.3% to 16.4%. Several observations are noteworthy:

LightRAG benefits most. LightRAG, which relies solely on pure semantic retrieval, shows the largest relative improvement (16.1% Factual, 16.4% Contextual). This suggests that our preprocessing modules effectively address the inherent weaknesses of pure semantic approaches, providing complementary entity-precision capabilities.

Structured frameworks show steady gains. GraphRAG, which incorporates graph-based structures, achieves consistent improvements. This indicates that our modules can be synergistically combined with structured retrieval paradigms.

General applicability validated. The universal improvement across diverse RAG architectures confirms that our “conservatism” and “symmetry” design philosophy addresses fundamental limitations of entity-intensive retrieval, not framework-specific artifacts.

### 4.8 HRRF parameter sensitivity analysis (E6)

To validate the robustness of our HRRF module and guide practical implementation, we performed a sensitivity analysis on two key parameters: (1) the RRF smoothing constant δ, and (2) the BM25/Dense weighting ratio in alternative weighted-sum fusion.

**Figure 3:**
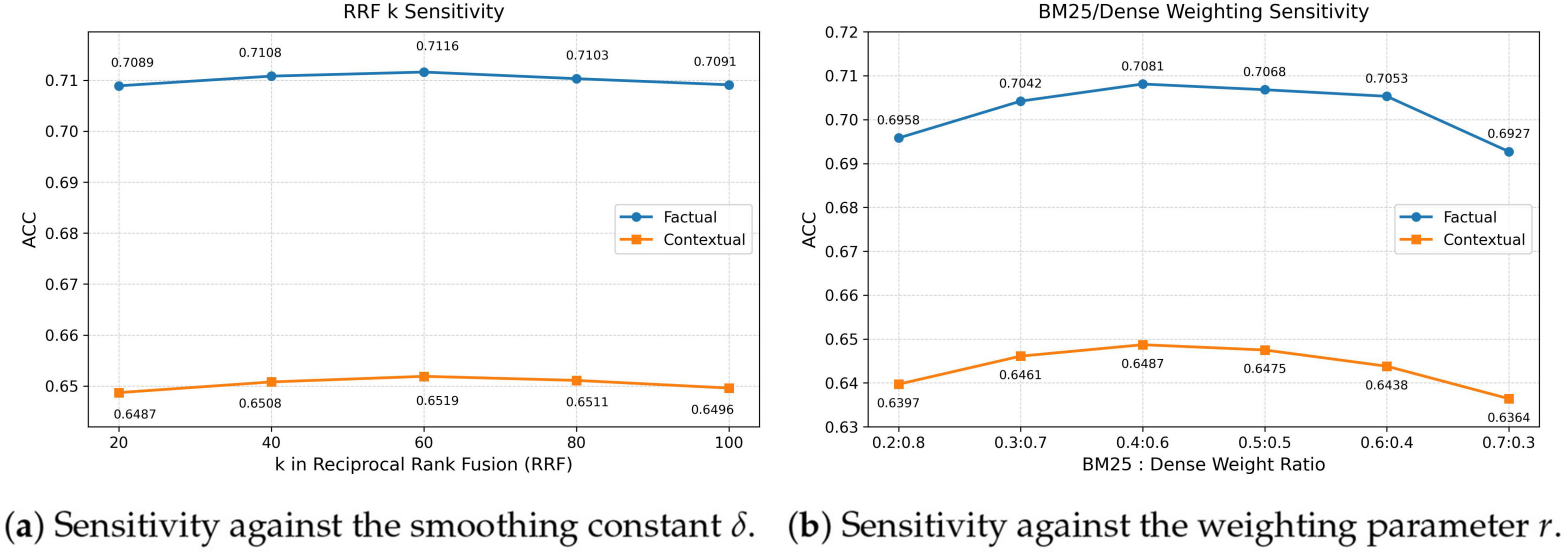
Parameter sensitivity analysis of the HRRF module. Both the RRF smoothing constant δ and the BM25-to-dense weight ratio *r* influence retrieval accuracy within a bounded range, yet the overall performance remains stable with minimal fluctuation, demonstrating the robustness of our fusion strategy. **(a)** Sensitivity against the smoothing constant δ. **(b)** Sensitivity against the weighting parameter *r*.

As shown in Figure 3, the sensitivity analysis yields several practical insights:

RRF is robust to parameter selection. Performance variation across δ values is minimal, confirming that RRF's rank-based fusion is inherently stable. The default δ = 60 provides optimal performance, consistent with prior literature (Cormack et al., 2009).

Balanced weighting is preferred. The optimal BM25:Dense ratio is 0.4:0.6, slightly favoring dense retrieval. However, the performance gap between balanced and optimal weighting is negligible, indicating robustness to configuration choices.

RRF outperforms weighted fusion. Notably, the best RRF result (0.7116, δ = 60) exceeds the best weighted-sum result, validating RRF's theoretical advantages in fusing heterogeneous retrieval signals.

### 4.9 Cost-benefit analysis (E7)

To assess the practical deployability of our method, we conduct a comprehensive cost-benefit analysis comparing our approach against representative alternatives that require additional LLM inference: LLM Reranker (RankGPT) (Sun et al., 2023) and HyDE. We measure both computational cost (token consumption) and effectiveness gain. Tables 7, 8 present the results of the cost analysis.

**Table 7:** Token consumption comparison.

Method	Offline tokens	Online tokens/query	Online total (1,000 q)	Total extra tokens
Ours (CQR+ESCA)	1,485,471	389	127,291	1,612,762
LLM Reranker (RankGPT, Sun et al., 2023)	0	8,285	8,285,127	8,285,127
HyDE	0	247	247,025	247,025

**Table 8:** Cost-efficiency comparison (Factual ACC).

Method	ACC	Δ vs. baseline	Online tokens (1,000 q)	Tokens/ACC point
Ours (CQR+ESCA)	**0.7348**	**+0.0605**	127,291	**2,104**
LLM Reranker	0.7185	+0.0442	8,285,000	187,443
HyDE	0.6480	–0.0263	247,000	Negative ROI

The bold values indicate the best performance among the compared algorithms.

The cost-benefit analysis reveals a compelling advantage:

Ours achieves best cost-efficiency. Our method requires only 2,104 tokens per ACC point improvement, compared to 187,443 for LLM Reranker—an 89 × improvement in cost-efficiency. This dramatic advantage stems from our offline preprocessing strategy: ESCA is a one-time cost (1.49M tokens) amortized across all queries, while CQR's online cost (389 tokens/query) is minimal compared to LLM Reranker's 8,285 tokens/query.

HyDE has negative ROI. Despite its low per-query cost (247 tokens), HyDE degrades performance, resulting in negative return on investment. This confirms that aggressive query expansion is not only ineffective but counterproductive in entity-intensive music retrieval.

Offline investment pays off. The 1.49M offline tokens invested in ESCA enable substantial online savings. For a system serving 1,000 queries, our method's total extra tokens are only 19% of LLM Reranker's online cost alone, while achieving superior performance.

## 5 Discussion

This work contributes to the growing body of research on adapting general-purpose RAG systems to domain-specific challenges. By demonstrating that substantial improvements can be achieved through thoughtful system design without model retraining or complex knowledge graph construction, this work suggests that practical and cost-effective solutions remain viable for addressing retrieval challenges in specialized domains.

However, this work has several limitations that warrant future investigation. First, our evaluation was conducted in only the music scenario (Kwon et al., 2025); the generalization of findings to other entity-intensive domains such as healthcare (Xiong et al., 2024), law (Wiratunga et al., 2024) or e-commerce (Benita et al., 2024) remains to be validated. Second, the ESCA and CQR modules rely on LLM calls, which introduce latency and cost; while our cost-benefit analysis showed favorable efficiency, the dependency on external API calls may limit deployment in offline or resource-constrained environments. Third, our current approach focuses on single-hop entity retrieval; the extension to multi-hop reasoning scenarios where entities must be connected through intermediate relations remains an open challenge.

Future directions include investigating domain-adaptive strategies that automatically adjust the degree of query rewriting based on entity frequency estimates, exploring lightweight alternatives to LLM-powered modules for resource-limited settings, and integrating the proposed framework with knowledge graph structures (Granata et al., 2025) to support complex multi-hop queries in music knowledge bases.

## 6 Conclusions

In this work, we identify semantic drift as the main cause of long-tail entity retrieval failure in question answering tasks. Based on this observation, we propose the HCE-RAG framework, which effectively addresses this pain point through a systematic integration of three orthogonal enhancement mechanisms. Our experiments and analysis yield three main findings: (1) minimal necessary rewriting successfully avoids hallucinatory noise and outperforms aggressive expansion; (2) Entity-Sensitive Contextual Augmentation (ESCA) enhances the visibility of low-frequency entities in the vector space at low cost; and (3) the symmetric interplay between ESCA and CQR robustly bridges the indexing and query sides, thereby maximizing hybrid retrieval effectiveness.

We emphasize that HCE-RAG does not propose an entirely new algorithmic paradigm. Rather, it represents a systematic integration contribution: the hierarchical combination of ESCA, CQR, and HRRF modules, grounded in a conservatism and symmetry design philosophy specifically oriented toward entity-intensive vertical domains. This systematic coordination—where each module addresses a complementary aspect of the retrieval pipeline—enables plug-and-play enhancement for existing RAG systems without requiring model retraining or knowledge graph construction. Our framework demonstrates that in precision-critical entity-intensive scenarios, architectural discipline and systematic coordination often outweigh simply choosing larger models or scaling up computation.

## Data Availability

The original contributions presented in the study are included in the article/supplementary material, further inquiries can be directed to the corresponding author.

## References

[B1] Benita J. Tej K. V. C. Kumar E. V. Subbarao G. V. Venkatesh C. (2024). “Implementation of retrieval- augmented generation (rag) in chatbot systems for enhanced real-time customer support in e- commerce,” in Proceedings of the 2024 3rd International Conference on Automation, Computing and Renewable Systems (ICACRS) (IEEE), 1381–1388. doi: 10.1109/ICACRS62842.2024.10841586

[B2] Bhagdev R. Chapman S. Ciravegna F. Lanfranchi V. Petrelli D. (2008). “Hybrid search: Effectively combining keywords and semantic searches,” in Proceedings of the European Semantic Web Conference (Berlin, Heidelberg: Springer), 554–568. doi: 10.1007/978-3-540-68234-9_41

[B3] Cormack G. V. Clarke C. L. Buettcher S. (2009). “Reciprocal rank fusion outperforms condorcet and individual rank learning methods,” in Proceedings of the Proceedings of the 32nd International ACM SIGIR Conference on Research and Development in Information Retrieval (New York, NY: Association for Computing Machinery), 758–759. doi: 10.1145/1571941.1572114

[B4] Cui W. Bi K. Guo J. Cheng X. (2024). “MORE: multi-mOdal REtrieval augmented generative commonsense reasoning,” in Proceedings of the Findings of the Association for Computational Linguistics: ACL 2024 (Bangkok: Association for Computational Linguistics), 1178–1192. doi: 10.18653/v1/2024.findings-acl.69

[B5] Edge D. Trinh H. Cheng N. Bradley J. Chao A. Mody A. . (2024). From local to global: a graph rag approach to query-focused summarization. arXiv preprint arXiv:2404.16130. Available online at: https://api.semanticscholar.org/CorpusID:269363075

[B6] Gao L. Ma X. Lin J. Callan J. (2023). “Precise zero-shot dense retrieval without relevance labels,” in Proceedings of the Proceedings of the 61st Annual Meeting of the Association for Computational Linguistics (Volume 1: Long Papers) (Toronto: Association for Computational Linguistics), 1762–1777. doi: 10.18653/v1/2023.acl-long.99 PMC12007664 40255468

[B7] Gao Y. Xiong Y. Gao X. Jia K. Pan J. Bi Y. . (2023). Retrieval-augmented generation for large language models: a survey. arXiv preprint arXiv:2312.109976199. Available online at: https://api.semanticscholar.org/CorpusID:266359151

[B8] Granata F. Poggi F. Mongiovi M. (2025). Enhancing retrieval-augmented generation with entity linking for educational platforms. Big data Cogn. Comput.

[B9] Guo Z. Xia L. Yu Y. Ao T. Huang C. (2025). “LightRAG: simple and fast retrieval-augmented generation,” in Proceedings of the Findings of the Association for Computational Linguistics: EMNLP 2025 (Suzhou: Association for Computational Linguistics), 10746–10761. doi: 10.18653/v1/2025.findings-emnlp.568

[B10] Gutierrez B. J. Shu Y. Qi W. Zhou S. Su Y. (2025). “From RAG to memory: non-parametric continual learning for large language models,” in Proceedings of the International Conference on Machine Learning (PMLR), 21497–21515.

[B11] Hu D. Dong Z. Liang K. Yu H. Wang S. Liu X. (2024). High-order topology for deep single-cell multi-view fuzzy clustering. IEEE Trans. Fuzzy Syst. 32, 4448–4459. doi: 10.1109/TFUZZ.2024.3399740

[B12] Hu D. Guan R. Dong Z. Liang K. Wang J. Wang S. . (2025). Single-cell multi-view clustering via community detection with unknown number of clusters. IEEE Trans. Comput. Biol. Bioinform. 23, 122–133. doi: 10.1109/TCBBIO.2025.3636975 41289140

[B13] Jagerman R. Zhuang H. Qin Z. Wang X. Bendersky M. (2023). Query expansion by prompting large language models. arXiv preprint arXiv:2305.03653. Available online at: https://api.semanticscholar.org/CorpusID:258546701

[B14] Karpukhin V. Oguz B. Min S. Lewis P. Wu L. Edunov S. . (2020). “Dense passage retrieval for open-domain question answering,” in Proceedings of the Proceedings of the 2020 Conference on Empirical Methods in Natural Language Processing (EMNLP) (Association for Computational Linguistics), 736769–6781. doi: 10.18653/v1/2020.emnlp-main.550

[B15] Kulkarni H. MacAvaney S. Goharian N. Frieder O. (2023). “Lexically-accelerated dense retrieval,” in Proceedings of the Proceedings of the 46th International ACM SIGIR Conference on Research and Development in Information Retrieval (New York, NY: Association for Computing Machinery), 152–162. doi: 10.1145/3539618.3591715

[B16] Kuo T. L. Chiu W. N. Ma W. Y. Cheng P. J. (2025). Doc2Query++: topic-coverage based document expansion and its application to dense retrieval via dual-index fusion. arXiv preprint arXiv:2510.09557. Available online at: https://api.semanticscholar.org/CorpusID:282056107

[B17] Kwon D. Doh S. Nam J. (2025). Must-rag: musical text question answering with retrieval augmented generation. arXiv preprint arXiv:2507.23334. Available online at: https://api.semanticscholar.org/CorpusID:280401332

[B18] Lewis P. Perez E. Piktus A. Petroni F. Karpukhin V. Goyal N. . (2020). “Retrieval-augmented generation for knowledge-intensive NLP tasks,” in Proceedings of the 34th International Conference on Neural Information Processing Systems (Red Hook, NY: Curran Associates Inc.).

[B19] Mackie I. Chatterjee S. Dalton J. (2023). “Generative relevance feedback with large language models,” in Proceedings of the 46th international ACM SIGIR Conference on Research and Development in Information Retrieval (New York, NY: Association for Computing Machinery), 2026–2031. doi: 10.1145/3539618.3591992

[B20] Mao Y. He P. Liu X. Shen Y. Gao J. Han J. . (2021). “Generation-augmented retrieval for open-domain question answering,” in Proceedings of the Proceedings of the 59th Annual Meeting of the Association for Computational Linguistics and the 11th International Joint Conference on Natural Language Processing (Volume 1: Long Papers) (Association for Computational Linguistics), 4089–4100. doi: 10.18653/v1/2021.acl-long.316

[B21] Mao Z. Zhao M. Wu Q. Wakaki H. Mitsufuji Y. (2025). “Deepresonance: enhancing multimodal music understanding via music-centric multi-way instruction tuning,” in Proceedings of the Proceedings of the (2025). Conference on Empirical Methods in Natural Language Processing (Suzhou: Association for Computational Linguistics), 12937–12959. doi: 10.18653/v1/2025.emnlp-main.653

[B22] Min D. Zhang K. Wu T. Cheng L. QuCo-RA G. (2025). Quantifying uncertainty from the pre- training corpus for dynamic retrieval-augmented generation. arXiv preprint arXiv:2512.19134. Available online at: https://api.semanticscholar.org/CorpusID:284077975

[B23] Nogueira R. Yang W. Lin J. Cho K. (2019). Document expansion by query prediction. arXiv preprint arXiv:1904.08375.

[B24] Qiu Y. Li H. Qu Y. Chen Y. She Q. Liu J. . (2022). “DuReader-retrieval: a large- scale chinese benchmark for passage retrieval from web search engine,” in Proceedings of the Proceedings of the 2022 Conference on Empirical Methods in Natural Language Processing, eds. Y. Goldberg, Z. Kozareva, and Y. Zhang (Abu Dhabi: Association for Computational Linguistic), 5326–5338. doi: 10.18653/v1/2022.emnlp-main.357

[B25] Robertson S. Zaragoza H. (2009). The probabilistic relevance framework: BM25 and Beyond. Inf. Retr. 3, 333–389.

[B26] Sun W. Yan L. Ma X. Wang S. Ren P. Chen Z. . (2023). “Is ChatGPT good at search? Investigating large language models as re-ranking agents,” in Proceedings of the 2023 Conference on Empirical Methods in Natural Language Processing (Singapore: Association for Computational Linguistics), 14918–14937. doi: 10.18653/v1/2023.emnlp-main.923

[B27] Thakur N. Reimers N. Ruckle A. Srivastava A. Gurevych I. (2021). BEIR: a heterogeneous benchmark for zero-shot evaluation of information retrieval models. CoRR. abs/2104.08663. Available online at: https://arxiv.org/abs/2104.08663

[B28] Wang L. Yang N. Wei F. (2023). “Query2doc: query expansion with large language models,” in Proceedings of the Proceedings of the (2023). Conference on Empirical Methods in Natural Language Processing (Singapore: Association for Computational Linguistics), 9414–9423. doi: 10.18653/v1/2023.emnlp-main.585

[B29] Wiratunga N. Abeyratne R. Jayawardena L. Martin K. Massie S. Nkisi-Orji I. . (2024). “CBR-RAG: case-based reasoning for retrieval augmented generation in LLMs for legal question answering,” in Proceedings of the International Conference on Case-Based Reasoning (Cham: Springer), 445–460. doi: 10.1007/978-3-031-63646-2_29

[B30] Wu S. Zhancheng G. Yuan R. Jiang J. Doh S. Xia G. . (2025). “Clamp 3: universal music information retrieval across unaligned modalities and unseen languages,” in Proceedings of the Findings of the Association for Computational Linguistics: ACL 2025 (Vienna: Association for Computational Linguistics), 2605–2625. doi: 10.18653/v1/2025.findings-acl.133

[B31] Xiang Z. Wu C. Zhang Q. Chen S. Hong Z. Huang X. . (2025). When to use graphs in rag: a comprehensive analysis for graph retrieval-augmented generation. arXiv preprint arXiv:2506.05690. Available online at: https://api.semanticscholar.org/CorpusID:279244678

[B32] Xiong G. Jin Q. Lu Z. Zhang A. (2024). “Benchmarking retrieval-augmented generation for medicine,” in Proceedings of the Findings of the Association for Computational Linguistics: ACL 2024, eds. L. W. Ku, A. Martins, and V. Srikumar (Bangkok: Association for Computational Linguistics), 6233–6251. doi: 10.18653/v1/2024.findings-acl.372

[B33] Xu Z. Zhuang S. Ma X. Chen B. Tian Y. Mo F. . (2025). Rethinking On-policy optimization for query augmentation. arXiv preprint arXiv:2510.17139. Available online at: https://api.semanticscholar.org/CorpusID:282209069

[B34] Zhang J. Li C. Kosov S. Grzegorzek M. Shirahama K. Jiang T. . (2021). LCU- Net: A novel low-cost U-Net for environmental microorganism image segmentation. Pattern Recogn. 115:107885. doi: 10.1016/j.patcog.2021.107885

[B35] Zhang J. Li C. Yin Y. Zhang J. Grzegorzek M. (2023). Applications of artificial neural networks in microorganism image analysis: a comprehensive review from conventional multilayer per- ceptron to popular convolutional neural network and potential visual transformer. Artif. Intell. Rev. 56, 1013–1070. doi: 10.1007/s10462-022-10192-7 35528112 PMC9066147

[B36] Zhang J. Liu L. Gao K. Hu D. (2025a). A forward and backward compatible framework for few-shot class-incremental pill recognition. IEEE Trans. Neural Netw. Learn. Syst. 36, 9837–9851. doi: 10.1109/TNNLS.2024.3497956 40030571

[B37] Zhang J. Liu L. Silven O. Pietikainen M. Hu D. (2025b). Few-shot class-incremental learning for classification and object detection: a survey. IEEE Trans. Pattern Anal. Mach. Intell. 47, 2924–2945. doi: 10.1109/TPAMI.2025.3529038 40031007

[B38] Zhang J. Zhao P. Zhao Y. Li C. Hu D. (2025c). Few-Shot Class-Incremental Learning for Retinal Disease Recognition. IEEE J. Biomed. Health Inform. 29, 9001–9012. doi: 10.1109/JBHI.2024.3457915 39292587

[B39] Zhao Y. Fan Z. Cao Y. Lyu Z. Li J. (2026). RewriteGen: autonomous query optimization for retrieval-augmented large language models via reinforcement learning. Electronics 15:1058. doi: 10.3390/electronics15051058

[B40] Zheng H. S. Mishra S. Chen X. Cheng H. T. Chi E. H. Le Q. V. . (2023). Take a step back: evoking reasoning via abstraction in large language models. arXiv preprint arXiv:2310.06117.

[B41] Zheng H. S. Mishra S. Chen X. Cheng H. T. Chi E. H. Le Q. V. . (2024). “Take a step back: evoking reasoning via abstraction in large language models,” in Proceedings of the International Conference on Learning Representations, eds. B. Kim, Y. Yue, S. Chaudhuri, K. Fragkiadaki, M. Khan, and Y. Sun, Vol. 2024, 20279–20316. Available online at: https://proceedings.iclr.cc/paper_files/paper/2024/file/592da1445a51e54a3987958b5831948f-Paper-Conference.pdf

[B42] Zhuang H. Qin Z. Hui K. Wu J. Yan L. Wang X. . (2024). “Beyond yes and no: improving zero-shot llm rankers via scoring fine-grained relevance labels,” in Proceedings of the Proceedings of the 2024 Conference of the North American Chapter of the Association for Computational Linguistics: Human Language Technologies (Volume 2: Short Papers) (Mexico City: Association for Computational Linguistics), 358–370. doi: 10.18653/v1/2024.naacl-short.31

